# Emergence of Resistance to MTI-101 Selects for a MET Genotype and Phenotype in EGFR Driven PC-9 and PTEN Deleted H446 Lung Cancer Cell Lines

**DOI:** 10.3390/cancers14133062

**Published:** 2022-06-22

**Authors:** Clark Jones, Sebastian Dziadowicz, Samuel Suite, Ashley Eby, Wei-Chih Chen, Gangqing Hu, Lori A. Hazlehurst

**Affiliations:** 1 Department of Pharmaceutical Sciences, School of Pharmacy West Virginia University, Morgantown, WV 26505, USA; cj0035@mix.wvu.edu; 2 Department of Microbiology, Immunology and Cell Biology School of Medicine, West Virginia University, Morgantown, WV 26501, USA; sedziadowicz@mix.wvu.edu (S.D.); michael.hu@hsc.wvu.edu (G.H.); 3 Modulation Therapeutics Inc., Morgantown, WV 26506, USA; suite1@marshall.edu; 4 Cancer Institute, West Virginia University, Morgantown, WV 26501, USA; ane0013@mix.wvu.edu (A.E.); weichih.chen@hsc.wvu.edu (W.-C.C.)

**Keywords:** MET 1, invasion 2, EGFR 3, TWIST 4, drug resistance 5

## Abstract

**Simple Summary:**

MTI-101 is a first-in-class novel cyclic peptide shown to have anti-tumor activity in both multiple myeloma and castrate-resistant prostate in vivo cancer models. These data suggest the potential for a broad spectrum of anti-cancer activity for this class of compounds. To further delineate determinants of sensitivity and resistance that were not dependent on oncogenic drivers, two isogenic drug-resistant cell lines were generated with chronic exposure to MTI-101 in a non-small cell lung cancer (NSCLC) PC-9 (EGFR driven) and a small cell lung cancer (SCLC) H446 (PTEN deleted and c-MYC amplified). Our data indicate that the chronic exposure of MTI-101 selects for a stable mesenchymal-to-epithelial (MET) genotype and phenotype in both PC-9 and H446 lung cancer cell lines.

**Abstract:**

MTI-101 is a first-in-class cyclic peptide that kills cells via calcium overload in a caspase-independent manner. Understanding biomarkers of response is critical for positioning a novel therapeutic toward clinical development. Isogenic MTI-101-acquired drug-resistant lung cancer cell line systems (PC-9 and H446) coupled with differential RNA-SEQ analysis indicated that downregulated genes were enriched in the hallmark gene set for epithelial-to-mesenchymal transition (EMT) in both MTI-101-acquired resistant cell lines. The RNA-SEQ results were consistent with changes in the phenotype, including a decreased invasion in Matrigel and expression changes in EMT markers (E-cadherin, vimentin and Twist) at the protein level. Furthermore, in the EGFR-driven PC-9 cell line, selection for resistance towards MTI-101 resulted in collateral sensitivity toward EGFR inhibitors. MTI-101 treatment showed synergistic activity with the standard of care agents erlotinib, osimertinib and cisplatin when used in combination in PC-9 and H446 cells, respectively. Finally, in vivo data indicate that MTI-101 treatment selects for increased E-cadherin and decreased vimentin in H446, along with a decreased incident of bone metastasis in the PC-9 in vivo model. Together, these data indicate that chronic MTI-101 treatment can lead to a change in cell state that could potentially be leveraged therapeutically to reduce metastatic disease.

## 1. Introduction

Lung cancer is the deadliest of all cancers and accounts for more deaths each year than breast, prostate and colon cancer combined [1]. Upon diagnosis, lung cancer can be categorized into two subtypes: small cell lung cancer (SCLC) and non-small cell lung cancer (NSCLC). Almost all SCLC patients are current or former tobacco smokers, and cases of SCLC typically have very aggressive tumors grouped with a poor five-year survival rate below seven percent [2]. There are few treatment options for SCLC patients because many of the identified molecular drivers are not currently druggable, along with the presence of considerable tumor heterogeneity, which likely contributes to the rapid emergence of drug-resistant disease. The emergence of resistance and metastatic disease contributes to the 5-year survival rate of SCLC patients at a dismal 7%. In contrast, NSCLC patients include smokers and non-smokers. In fact, 10–15% of all newly diagnosed NSCLC patients have never smoked [3]. Many therapeutically actionable mutational drivers have been identified in this class of lung cancer, such as EGFR and ALK mutations, that allow for targeted therapies for these subsets of lung cancer patients. Despite advances in targeted therapeutics, metastatic NSCLC remains a deadly disease due to the rapid emergence of drug resistance. Recent data indicate that one way to combat drug resistance is to design therapeutic strategies that keep a drug-sensitive population available for further treatment. Thus, considering compounds that have non-overlapping drug-resistant profiles and alternating the schedule as single agents may lead to improved patient outcomes by delaying the emergence of an aggressive drug-resistant phenotype [4]. 

MTI-101 is a first-in-class novel therapeutic agent that was originally derived from a linear peptide known as HYD-1, found in a high throughput screen that utilized the inhibition of cell adhesion as a phenotypic endpoint [5]. It was later found to induce cell death in cancer cells by altering calcium homeostasis in a caspase-independent manner [6]. Experimental evidence indicates that MTI-101 selectively targets cancer cells via the activation of a TRPC complex and calcium entry, leading to cell death [7,8]. MTI-101 has shown strong activity in relapsed multiple myeloma primary patient specimens tested ex-vivo and castrate-resistant prostate in vivo models by reducing the tumor volume and increasing survival, with little observed toxicity at the efficacious doses used for in vivo studies [7,9]. Interestingly, resistance to HYD-1 in myeloma cell lines resulted in a compromised cell-adhesion-mediated resistant phenotype, suggesting that resistance emerged at an overall cost to the cell, related to cell adhesion [10]. We hypothesized that this cyclized peptide known as MTI-101 may be an attractive therapeutic agent for metastatic lung cancer, as calcium signaling pathways are critical for metastasis [11]. The identification of phenotypic traits and changes in states due to genotypic changes that occur with the acquisition of resistance to MTI-101 will enhance the understanding of how to clinically position this first-in-class molecule. 

The primary site of a tumor will eventually be limited by resources, which places an initial selection pressure to favor cells that are capable of invading the surrounding tissue or relocating to additional regions in the body to obtain the necessary nutrients for the survival of rapidly dividing cells. This cellular rearrangement, called epithelial-to-mesenchymal transition (EMT), is found to take place in normal cells for tasks such as development and wound healing, but cancer cells can undergo these changes in response to stress stimuli [12,13,14]. This transition allows for epithelial-like tumor cells to alter gene expression, such as decreasing epithelial proteins, including E-cadherin, claudin and occludin, while increasing mesenchymal proteins such as vimentin (VIM), N-cadherin and fibronectin (FN1), allowing them to survive after losing contact from adjacent cells and the basement membrane [15]. EMT cells can undergo cytoskeleton rearrangement to become more motile and can also produce proteins such as matrix metalloproteases (MMPs) that allow them to invade into new tissues and metastasize [16,17]. Clinically, malignant cells displaying an EMT phenotype after drug treatment correspond with a poor prognosis that leads to a decreased relapse time and increased metastatic tumor sites [18,19,20]. This transition is transient, and cells can also change to a more epithelial-like phenotype moving toward MET [21]. Patients that are treated with current standard of care therapies for lung cancer often experience relapse, which gives rise to drug-resistant tumors that have undergone EMT [20]. Recent studies have shed light on EMT as a spectrum of cell states compared to a binary process [22]. Early models supported the claim of the existence of hybrid EMT models by developing mathematical equations to describe a unique model of the microRNA-based coupled chimeric modules that utilize ZEB and Snail mutual inhibition feedback circuits [23]. This phenomenon was further explained by Snail microRNA serving as a reversible switch to initiate EMT, while ZEB microRNA acts as an irreversible switch to establish the mesenchymal state with the interplay of TGF-β signaling [24]. More recent in vivo studies have shown that, due to the heterogeneity of tumor populations, there are six distinct EMT subpopulations that exist based on cell surface markers that differentiate cellular plasticity, invasiveness and metastatic potential [25]. Due to the unique mechanism of action of MTI-101, we sought to further understand the emerging drug-resistant phenotype using in vitro and in vivo models. The emergence of resistant cell lines was analyzed in comparison to the wildtype (WT) to determine the genotype and corresponding phenotype that the drug is selecting for in lung cancer. These data may allow for the identification of new actionable vulnerabilities that emerge with chronic selection pressure of MTI-101 treatment that may inform rational therapeutic strategies to be further validated in pre-clinical lung cancer models prior to the development of clinical strategies.

## 2. Materials and Methods

### 2.1. Cell Culture/Reagents

PC-9 (Sigma #90071810) H446 (ATCC #HTB-171) and HCC4006 (ATCC #CRL-2871) lung cancer cell lines were used as parental cell lines. MTI-101 resistant cell lines PC-9R and H446-R were developed with chronic exposure to MTI-101 over a 6-month period. All cell lines were maintained in RPMI-1640 L-glutamine with 10% fetal bovine serum and 1% penicillin/streptomycin. Resistant cell lines were maintained with a constant level of MTI-101 in culture media (60 µM for PC-9R and 40 µM for H446-R). All cell lines were tested for mycoplasma every six months, and STTR analysis was performed on a yearly basis. 

### 2.2. MTT Assays

PC-9 cells were seeded at 10,000 cells/180 µL media and H446 was seeded at 20,000 cells/180 µL media in each well of a 96-well plate. The following day, 20 µL of varying drug concentrations of MTI-101 dissolved in sterile water was added to each treatment group, with 20 µL of sterile water added to the controls. Twenty-four hours after the introduction of MTI-101, 50 µL of MTT dye was added to each well and incubated for one hour. Following incubation, the media/dye were aspirated off, the cells were resuspended in 200 µL of DMSO and the absorbance of each well was read on a plate reader at 570 nm. A Log_10_ (Dose) curve was generated using GraphPad to calculate the IC_50_ values of each cell line. 

### 2.3. Western Blots

All cells were lysed in buffer composed of 50 mm Tris (pH 7.4), 5 mm EDTA, 150 mm NaCl and 0.5% Triton X-100 containing 1 μg/mL leupeptin and aprotinin and 1 mm phenylmethyl sulfonyl fluoride. The protein content of cell lysates was quantified by using the Pierce BCA protein assay kit, and equal amounts of the total protein were dissolved in Laemmli SDS-PAGE sample buffer prior to separation by 15% SDS-PAGE. Proteins were transferred to polyvinylidene difluoride membrane (Bio-Rad), and Western blot analysis was performed by using standard techniques with enhanced chemiluminescence detection (Roche/Boehringer Mannheim, Indianapolis, IN, USA). The following antibodies were used in this experiment: E-cadherin, vimentin, B-catenin, TWIST1, B-actin (Cell Signaling, Cat #-3195T, 5741T, 8480T, 46702, 4967S) and TWIST2 (ThermoFisher 66544-1-IG).

### 2.4. Invasion Assay

PC-9 cells were seeded at 25,000 cells/mL media and H446 cells were seeded at 40,000 cells/mL. A total of 100 µL was dispensed in each well of a 96-well round bottom plate. Spheroids were allowed to form for 48 h. Then, 70 µL media were slowly aspirated off and the plate was placed on ice for 5 min. Next, 70 µL of Matrigel was slowly added on top of each spheroid and placed in the incubator to allow for gel solidification for 1 h. Following incubation, each well was topped with 100 µL media to prevent the gel from drying out, and the plate was placed on the Biotek Lionheart imager to track invasion over a 48 h period. The image montage was stitched and the most in focus z-parameter was selected for each time point. Invasion distance was quantified by creating a primary mask on the original spheroid at Time 0 and tracked over time divided by the Time 0 mask for the duration of the invasion assay.

### 2.5. Anoikis Assay

Apoptosis assays for anoikis were performed by assaying annexin v/pI staining. A total of 500,000 cells (50,000 cells/mL) were placed in 100 mm ultralow attachment dish (0.5% methyl cellulose; Sigma-Aldrich) for 24 h. The apoptotic cells were determined using propidium iodide (pI, 2 µg/mL; Thermo Fisher) and annexinv V-APC (1 µg/mL; BD Biosystems) using a FACScan flow cytometer (BD biosystems).

### 2.6. RNA Extraction

Quadruplicates of RNA were extracted from each WT and resistant lines of PC-9 and H446 using RNeasy Plus Mini Kit (Qiagen, cat#74104). Two to three million cells were isolated in a dry pellet and resuspended, and RNA was initially extracted using Trizol, further purified using the Qiagen RNeasy Mini Protocol and stored at −80 degrees. The same RNA isolation procedure was performed for the PC-9 4, 5 and 6 months removed from drug (RFD). 

### 2.7. RNA-Seq Data Analysis

RNA-Seq libraries were sequenced at Marshall University Genomics Core with Illumina NextSeq 2000. Pair-end RNA-SEQ short reads were aligned to the whole human reference genome (hg38) by subread v2.0.1 [26]. The total number of reads aligned to transcripts at gene level were summarized by *featurecounts* from Rsubread v2.2.0 [27] with built-in RefSeq gene annotation. Gene expression level was quantified by RPKM (reads per kilobase of exon model per million mapped) [28]. We used edgeR v3.30.3 [29] to define differentially expressed (DE) genes with the following criteria: *p*-value < 0.05, fold change > 1.5 and CPM (count per million; log2) > 0. To determine the relationship between expression changes in PC-9 and H446 to their MTI-101-resistant counter parts, we employed gene set enrichment analysis through GSEA v4.0.2. We created three “rnk” files based on fold change in expression (treatment vs. baseline) for the following comparisons: PC-9R vs. PC-9, H446-R vs. H446 and PC-9 RFD vs. PC-9; a “rnk” file consists of one column for gene symbol and another for fold change in expression. The rnk file was then supplied as input to GSEA to determine the significance of a preferential expression for selected gene sets from MSigDB in treatment condition over the baseline, or vice versa. 

### 2.8. Transfections

PC-9 and H446 cells were transfected with a tomato red/LUC vector (pcDBA3.1 (+)/Luv2 = tdT, Addgene #32904) [30] by electroporation using an Amexa nucleofection kit and Amexa nucleofector instrument per manufacture’s recommendation.

### 2.9. In Vivo Animal Studies

(H446 tomato red/LUC) A single treatment MTI-101 study was conducted in SCID/BEIGE mice to understand the single agent effect of MTI-101 in lung cancer. The mice (6–8 weeks old male/female) were injected with 5 million H446 cells (transfected with tomato red/LUC) via tail vein, allowed to engraft for four weeks and imaged on IVIS for tumor location and progression. Fourteen (M/F) mice were separated into two groups, dividing tumor burden, location of tumor and gender evenly. One group was treated with 20mM histidine (vehicle control) whereas the other was treated with 10 mg/kg MTI-101 via IP injection three times a week for the duration of the study (200 days). Once mice met study endpoints per ACUC protocol, mice were euthanized, and kidneys were collected for further analysis. In the H446 model, cells were engrafted consistently in the kidneys and, thus, this organ was processed for further analysis. Vimentin, E-cadherin and β-catenin stains were completed on slices of these kidneys of both groups. (PC-9 tomato red/LUC) A similar study was also conducted using the EGFR Driven NSCLC PC-9. The mice (6–8 weeks old M/F) were injected with 2 million PC-9 cells (transfected with tomato red/LUC) via tail vein, allowed to engraft for three weeks and imaged on IVIS for tumor location and progression. Twenty-one mice were separated into two groups, dividing tumor burden, location of tumor and gender evenly. One group was treated with 20mM histidine (vehicle control) whereas the other was treated with 10 mg/kg MTI-101 via IP injection beginning 3 weeks post-tumor-injection for 12 weeks. Once mice met study endpoints per IACUC guidelines, they were euthanized, and lungs were collected for further analysis of MET markers, including vimentin (Ventana #790-2917), E-cadherin (Ventana #790-4497) and B-catenin (Cell Marque #224M-14). Lung tissue sections were also stained with H&E to confirm presence of tumor, and IHC was used to detect markers of MET. For IHC studies, tissues obtained from 6 mice from control and 6 mice from the MTI-101-treated cohort were analyzed. Fiji software was used to analyze mean intensity based on tumor area.

All in vivo study designs were approved by the West Virginia University ACUC board. 

## 3. Results

### 3.1. Development of Isogenic MTI-101 Resistant Lung Cancer Cell Lines

**Figure 1:**
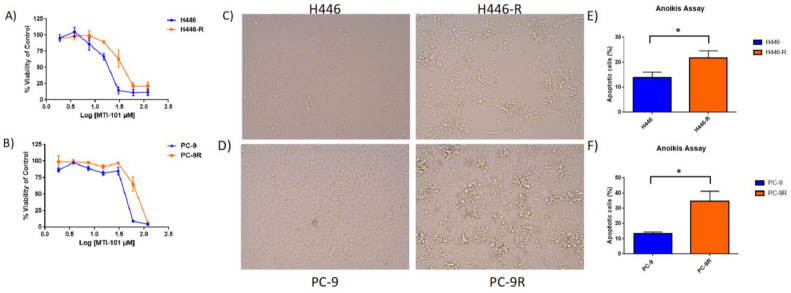
Development of isogenic MTI-101-resistant lung cancer cell lines. (**A**) Twenty-four-hour MTTs were performed with varying drug doses of MTI-101 comparing H446 to its respective resistant cell line. IC50 values were reported as an average *n*= 3 independent experiments. (**B**) Twenty-four-hour MTTs were performed with varying drug doses of MTI-101 comparing PC-9 to its respective resistant cell line. IC50 values were reported as an average *n* = 3 independent experiments. (**C**,**D**) Resistance to MTI-101 induces cell morphological changes to more adherent epithelial-like cells compared to the respective WT on a microscope at 20x magnification. (**E**,**F**) Anoikis assay comparing WT to respective resistant cell line for apoptotic percentage in low attachment plate. Results reported as *n* = 3. Error bars represent SEM (significances denoted * for *p* < 0.05, two-tailed *T*-test).

The development of isogenic-acquired drug-resistant cell lines is one strategy to identify determinants of sensitivity and resistance [31,32] to novel therapeutics. The isogenic cell lines allowed for paired analysis using RNA-SEQ to match the genotype to the emerging phenotype. PC-9 and H446 WT cell lines were initially treated with a low dose (10 µM) of MTI-101. The surviving or resistant cells were treated each subsequent time with 10 µM MTI-101 until they were able to proliferate at approximately the same rate as the parental WT cell line. The dose was then increased by 10 µM increments until a stable drug-resistant variant emerged. H446 was able to reach a 40 µM resistant level whereas PC-9 was stable up to a 60 µM resistant level. Interestingly, increasing the drug concentration beyond these doses was not achieved, as cultures did not sustain growth with an increased selection pressure. The stable variants were then compared to the respective WT cell line using an MTT analysis to assess resistant levels. The IC_50_ value shown is an average (*n* = 3 independent experiments) using the log dose of MTI-101 and viability compared to the control with non-linear regression on GraphPad. H446 and H446-R had, respectively, an IC_50_ value of 16.71 ± 3.97 and 34.51 ± 10.86 µM (Figure 1A). PC-9 and PC-9R had an IC_50_ value of 37.47 ± 4.33 and 82.11 ± 12.37 µM (Figure 1B). It was surprising to us that we could not select for higher levels of resistance, suggesting that a robust drug resistance phenotype may emerge at a high cost to overall fitness. In order to determine whether MTI-101 demonstrates specificity toward lung cancer cells, a healthy bronchial epithelial cell line known as BEAS-2B was also treated with the same range of doses of MTI-101 to determine its sensitivity. The IC_50_ value for BEAS-2B with treatment of MTI-101 was found to be 116.07 ± 29.05 µM. Together, these data indicate that lung cancer cells demonstrate an increased sensitivity compared to normal epithelial lung cells toward treatment with MTI-101. During the development of the two isogenic MTI-101-resistant lung cancer cell lines, morphological images were taken using bright field microscopy (20X), comparing each to their respective WT cell lines (Figure 1C,D). The resistant cell lines appeared to be more clumped and adherent to the culture dish than the WT that formed an even monolayer in vitro, suggesting increased cell–cell connections with resistance. This observation is suggestive of resistant cells converting to a more epithelial phenotype compared to the parental line. In parallel with morphological changes, resistant cells were also found to be significantly more sensitive to anoikis-induced apoptosis than their WT counterpart (Figure 1E,F).

### 3.2. Bioinformatic Analysis Discovers Enrichment of MET in Resistant Cell Lines

**Figure 2:**
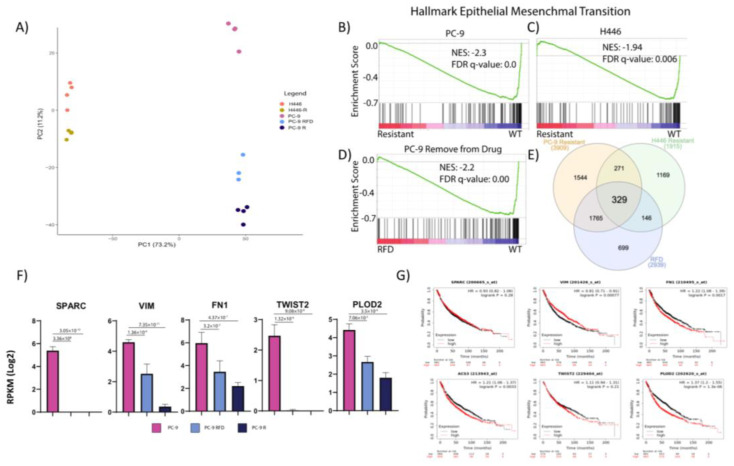
Transcriptome analysis indicated a downregulation in EMT in MTI-101-resistant lines. (**A**) PCA plot of all cell lines based on gene expression. (**B**–**D**) GSEA analysis of genes sorted based on fold changes in PC-9R vs. PC-9 (**B**), H446-R vs. H446 (**C**) or PC-9R RFD vs. PC-9 (**D**) from high (red) to low (blue) against MSigDB hallmark gene set “epithelial mesenchymal transition” (vertical bars). NES: normalized enriched score. (**E**) Venn diagram analysis of DE genes predicted for each resistance lines vs. corresponding WT. (**F**) Bar plot for expression values of SPARC, VIM, FN1, TWIST2 and PLOD2 across PC-9, RFD and PC-9R; FDR for differential expression from EdgeR indicated. (**G**) Individual genes (SPARC, VIM, FN1, ACS3/TWIST1, TWIST2 and PLOD2) split by median on all lung cancer patient data and predicted clinical prognosis in lung cancer. TWIST1 (ACS3), FN1 and PLOD2 are poor prognostic indicators for clinical outcome in lung cancer.

We applied RNA-seq analysis to determine differences in transcriptomes among PC-9 and H446 lung cancer cell lines and their MTI-101 resistant variants, PC-9R and H446-R, respectively. To determine the stability of changes in the transcriptome, the PC-9R line was removed from MTI-101 drug pressure and RNA was collected at four, five and six months post drug removal. PCA analysis based on gene expression revealed that replicates from the same cell lines were clustered together, confirming the high reproducibility of the data (Figure 2A).

The *x*-axis from the PCA plot depicts the cell type difference, and the *y*-axis demonstrates the expression difference between parental and MTI-101-resistant lines. The PC-9 RFD samples were closer to PC-9R than PC-9, indicating that removing the drug pressure for six months failed to convert the transcriptome of the resistant line back to its parental line. Gene set enrichment analysis was performed to determine pathways differentially expressed between WT and resistant cells. The results revealed that EMT is the most downregulated hallmark gene set in the MTI-101 resistance lines when compared to their parental lines (Figure 2B,C). Consistent with the PCA analysis, PC-9 RFD compared to PC-9 was also downregulated for the EMT gene set (Figure 2D), illustrating its similarity with PC-9R and the stability of the transcriptome signature after relieving drug pressure. A Venn diagram analysis was conducted to compare all of the differentially expressed genes between resistant cell lines and the WT lines (Figure 2E). It identified 329 genes commonly shared by all. Genes related to EMT in PC-9 were individually analyzed, including SPARC, vimentin, fibronectin, TWIST2 and PLOD2 (Figure 2F). Similar significant results were found in the negative expression of these EMT genes in the H446 cell line as well (Figure S1). In addition, these same genes were analyzed by relating their expression to the patient overall survival through the lung cancer Kaplan–Meier KM Plotter [33]. A low expression of TWIST1, fibronectin and PLOD2 predicted a significant increased patient survival (Figure 2G). In contrast, no changes in survival were observed based on the expression of SPARC or TWIST2 and the surprisingly low VIM expression as a single gene correlated with poor outcomes. Interestingly, EMT was negatively enriched in both resistant cell lines despite the vast differences in starting transcriptomes between the parental PC-9 and H446 with the sole selection pressure of MTI-101 treatment.

### 3.3. Emergence of Resistance to MTI-101 Selects for a Stable Phenotype Associated with Diminished Invasive Capacity and Increased Expression of Epithelial Markers

**Figure 3:**
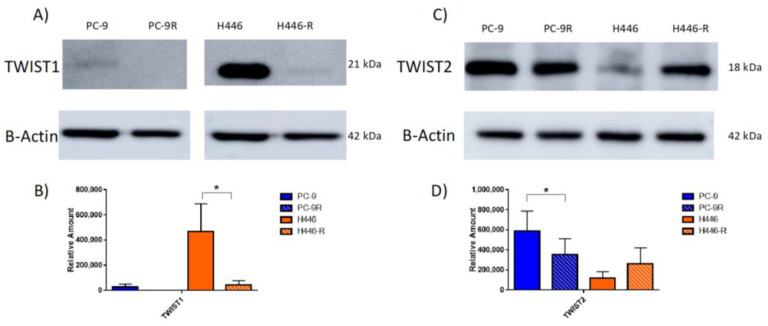
MTI-101-acquired drug-resistant lines demonstrate decreased expression of EMT transcription factor TWIST at the protein level. (**A**) Western blot probing for TWIST1 in WT and resistant cells; Western blot images were from the same blot but were spliced and rejoined between PC-9R and H446 samples in TWIST1 and β-actin images to remove protein standard marker for clarity. (**B**) Quantification of Western blot showing significant decreased protein expression of TWIST1 in H446-R cells compared to H446. (**C**) Western blot probing for TWIST2 in WT and resistant cells. (**D**) Quantification of Western blot showing significant decrease in protein expression of TWIST2 in PC-9-resistant cells compared to PC-9 WT. Completed as *n* = 3 independent experiments with reproducible representative images displayed for Western blots. Error bars represent SEM (significances denoted * for *p* < 0.05, two-tailed *T*-test).

Based on differential gene expression profiling, one of the only transcription factors shown to induce EMT that was differentially expressed in both cells lines was TWIST. Comparing parental to resistant lines, TWIST1 was significantly decreased in the H446-resistant variant (Figure 3A,B). In PC-9 cells, the basal level of TWIST1 is low and was further diminished in the resistant line (albeit the quantification of three blots did not show significance, *p* > 0.05 *T*-Test). In contrast, TWIST2 was significantly decreased in the PC-9 resistant variant (Figure 3C,D).

**Figure 4:**
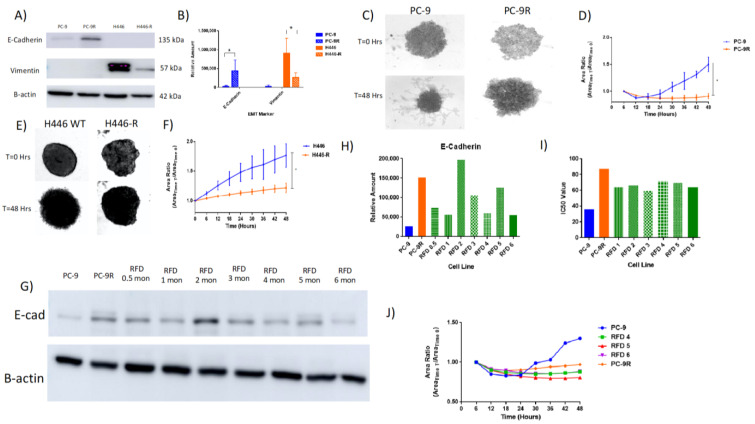
Emergence of Resistance to MTI-101 Selects for a Stable Phenotype Associated with Diminished Invasive Capacity and Increased Expression of Epithelial Markers. (**A**) PC-9 and H446 WT were compared to their respective MTI-101-resistant lines with respect to EMT protein markers. E-cadherin is an epithelial marker and was found to be overexpressed in the PC-9R cell line compared to WT. Vimentin is a mesenchymal marker and was found to be substantially decreased in H446-resistant cells compared to WT. Both corroborate a transition to a more epithelial-like cell phenotype (MET). (**B**) Quantification of Western blot. (**C**) PC-9 invasion assay comparing the WT to resistant cells’ ability to invade into the Matrigel over a 48 h time period. (**D**) Quantification of PC-9 invasion. (**E**) H446 invasion assay comparing the WT to resistant cells’ ability to invade into the Matrigel over a 48 h time period. (**F**) Quantification of H446 invasion. (**G**) Western blot comparing E-cadherin expression when PC-9R is removed from drug pressure over a 6-month period. (**H**) Quantification of Western blot for E-cadherin in PC-9 removed from drug (RFD). (**I**) IC_50_ values of MTTs run with MTI-101 treatment comparing PC-9 to its respective resistant and RFD cells. (**J**) Invasion assay showing lack of invasion of PC-9 RFD even after six months compared to PC-9 wild type cell line. Completed as *n* = 3 independent experiments, reproducible representative images shown for invasion images and Western blots. Error bars represent SEM (significances denoted * for *p* < 0.05, two-tailed *T*-test).

The original Western blots for individual runs are contained in the Figures S2–S4 for TWIST1 and Figures S5–S7 for TWIST2. The TWIST2 expression in H446-R did appear to trend towards an increased expression compared to H446, but was not found to be significant (*p* > 0.05, *T*-test *n* = 3). It is important to note that even though this difference is only two-fold, it could be compensating for a decreased TWIST1 expression in H446. For comparison, there was a ten-fold decrease in TWIST1 expression whereas only approximately a two-fold increased expression of TWIST2 comparing H446 to H446-R. In addition, the difference among resistant and sensitive cell lines was analyzed based on common EMT markers by Western blot analysis. PC-9 WT was found to have low E-cadherin levels, whereas PC-9R demonstrated a significantly increased expression (three-fold change) of E-cadherin at the protein level (Figure 4A,B). 

On the other hand, H446 WT was found to have a high expression of vimentin, whereas H446-R demonstrated a reduced expression by three-fold (Figure 4A,B). In addition, the invasion capacity was also measured for these cell lines. In both cases, the WT was found to have a significantly greater invasion capability in Matrigel over a 48 h period than that of the resistant line (Figure 4C–F).

The development of each MTI-101-resistant cell line required approximately six months of constant drug pressure with gradual increased increments of dosing before a stable drug resistant line emerged. In order to determine the stability of this acquisition of a MET phenotype, the drug pressure of MTI-101 was removed from the resistant cells and analyzed over a six-month span. The epithelial marker E-cadherin was found to not fully revert back to the expression level of the WT after six months of being removed from the drug (RFD) by Western blot analysis (Figure 4G,H). The original Western blots for individual runs can be found in Figures S8–S10 for Figure 4A along with Figure S11 for Figure 4G. Sensitivity to MTI-101 was analyzed by MTT comparing time from RFD compared to PC-9 WT and PC-9R. The IC_50_ values of RFD obtained a slight increase in sensitivity from their original PC-9R, but never fully reverted to the PC-9 WT sensitivity at each time point for the six months tested (Figure 4I). The inability to invade into Matrigel was compared among PC-9 RFD 4, 5 and 6 months to the PC-9 WT and PC-9R. Similarly, the invasion capacity of PC-9 RFD demonstrated a stable MET phenotype when the drug pressure was removed for six months (Figure 4J). Together, these data indicate that MTI-101 selects in vitro for a genotype and phenotype that are predicted to be less invasive.

### 3.4. MTI-101 Treatment In Vivo Recapitulates the In Vitro MET Phenotype

**Figure 5:**
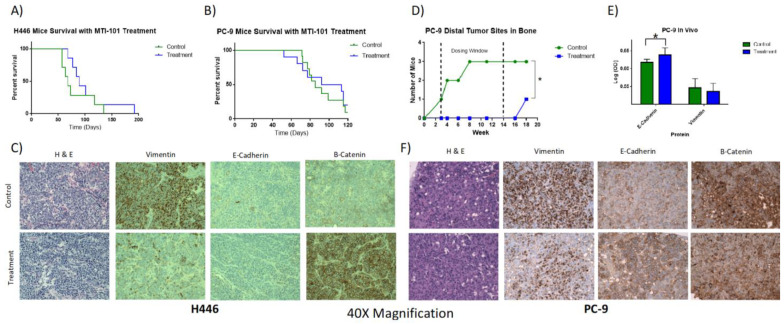
MTI-101 Treatment In Vivo Recapitulates the In Vitro MET Phenotype. Kidneys were taken from the H446 in vivo study, fixed in formalin, paraffin blocked and then sliced for slides to analyze EMT markers. The lungs of the PC-9 study went through the same process. Three control and three treatment (10 mg/kg MTI-101 three times per week IP injection) mice were picked at random from H446 and six of each in PC-9 were picked to compare EMT markers. (**A**) Survival curve shows no significant differences between MTI-101 treatment and control (*n* = 14, *p* > 0.05 log rank test). (**B**) Survival curve shows no significant differences between treatment and control in PC-9 study (*n* = 21, *p* > 0.05 log rank test). (**C**) H446 study tumor portions of the kidneys stained with H&E to confirm presence of tumor and EMT markers (vimentin, E-cadherin and β-catenin) demonstrates in vivo tumor transformation to MET with chronic treatment with 10 mg/kg of MTI-101. (**D**) PC-9 study quantity of mice detected with tumor in the bone over time course of study detected by IVIS and confirmed by H&E stains. (**E**) Quantification of E-cadherin and vimentin in IHC stains of PC-9 study (*n* = 6 tissues stained from control and drug treated cohort). (**F**) PC-9 H&E and IHC stains of lungs show presence of tumor and a significant increase in E-cadherin expression but no significant change in vimentin or β-catenin between control and MTI-101 treatment. Average representative image shown for all IHC. Tumors engrafted for 4 weeks (H446) and 3 weeks (PC-9) prior to drug treatment. Error bars represent SEM (significances denoted * for *p* < 0.05, two-tailed *T*-test).

In vivo animal models were conducted to determine whether MT-101 treatment in vivo induces an MET phenotype. The first study entailed the H446 WT tomato red/LUC cells being administered via the tail vein in SCID/BEIGE mice. Once mice met the study endpoints per IACUC protocol, they were euthanized, and the kidneys were extracted for further analysis. The kidneys were used for to stain for markers of MET in this model because it was the most common sight of engraftment for all mice in the study, while the lungs had little discernable tumor burden. The median survival of the treatment group was 89 days, compared to 68 days for the control, yet was not significant (*p*-value = 0.13 Figure 5A).

The H&E stains at 40× magnification confirmed that the cancerous portion of the kidney was being analyzed in these images for both the treatment and control. IHC was used to compare EMT markers. Vimentin was found to be greatly decreased whereas the membrane localization of β-catenin was substantially increased in the treatment group (Figure S12A,B), indicating that the treatment with MTI-101 was consistent with the transitioning of the cell state toward an epithelial phenotype (Figure 5C). E-cadherin was found to not be expressed in either group, which was consistent from the Western blots in vitro for this cell line. The 4× images of three mice from each group analyzed are shown in (Figure S13). 

PC-9 cells constitutively expressing tomato red and luciferase were injected via the tail vein into SCID/BEIGE mice. The two groups consisted of vehicle control and 10 mg/kg MTI-101 treatment three times a week for the duration of the study. A representative IVIS image for the engraftment of the study is shown in Figure S14. PC-9 engraftment was detected in the lungs of all animals in the study. IVIS imaging indicated that the detectable distal site of tumor outside of the lung was found in the hind limbs. These multiple tumor sites were disproportionate between control and drug treatment cohorts during the treatment window, with three mice containing tumors in the bone within the control group and zero in the treatment group (Figure 5D), along with significant increases in E-cadherin expression despite no significant change in vimentin or survival with treatment of MTI-101 compared to the control (Figure 5B,E,F). Following the end of the study, only one mouse in the MTI-101 treatment group was found to have tumor in the bone. Together, these data indicate that, at a dose of 10 mg/kg administered three times a week, although there was no significant difference in survival between treatment and control groups, the drug treatment was shown to induce a similar change in cell state seen in vitro towards a MET phenotype.

### 3.5. MTI-101′s Effect in Context with Standard of Care Treatment for Lung Cancer

**Figure 6:**
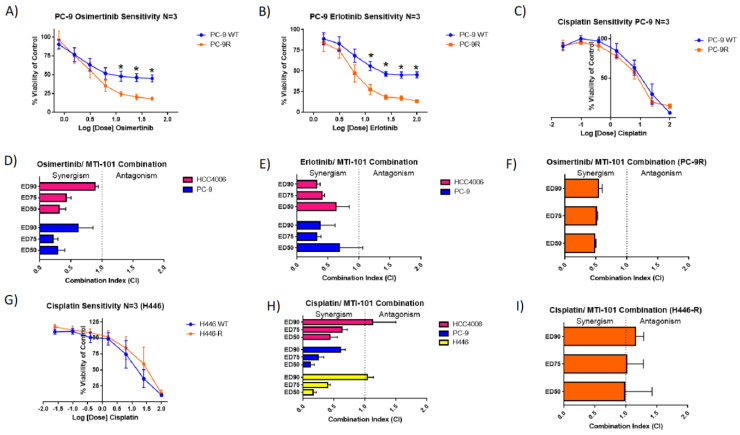
Selection for resistance to MTI-101 demonstrates collateral sensitivity to EGFR inhibitors but not Cis-Platinum treatment. Seventy-two-hour MTT assays were performed with the single treatment of osimertinib, erlotinib or cisplatin comparing WT to their respective MTI-101-resistant cell line. IC_50_ values comparing PC-9 to PC-9R were significant with osimertinib and erlotinib (significance *p* < 0.05, *T*-test) (**A**–**C**,**G**). Seventy-two-hour MTTs using combination therapy with MTI-101 in PC-9, HCC4006 and PC-9R demonstrate synergism with EGFR inhibitors (**D**–**F**). Forty-eight-hour MTTs using combination therapy with MTI-101 and cisplatin were synergistic in HCC4006, PC-9 and H446 cells (**H**) and additive in H446-R (**I**). Drug combination effects were determined by using CompuSyn software analysis. Shown is the mean CI of *n* = 3 independent experiments. Error bars represent SEM (significances denoted * for *p* < 0.05, two-tailed *T*-test).

PC-9 cells are driven by an exon 19 deletion causing EGFR to be constitutively active [34]. The identification of activating mutations has led to the discovery of EGFR inhibitors, including the first generation tyrosine kinase inhibitor erlotinib and third generation tyrosine kinase inhibitor targeting an additional T790M mutation osimertinib [35]. PC-9R cells were found to have a significantly increased sensitivity to osimertinib and erlotinib single treatment than that of the parental PC-9 (Figure 6A,B). 

The IC_50_ values of the PC-9 WT were 11.59 ± 0.09 and 25.91 ± 0.08 compared to the PC-9R of 4.57 ± 0.07 and 6.60 ± 0.06 nM, respectively, showing over a two-fold increase in sensitivity with both EGFR inhibitors. There was found to be no significant difference in sensitivity to cisplatin comparing PC-9 to PC-9R (Figure 6C). These data indicate that selection for resistance to MTI-101 drives collateral sensitivity to EGFR inhibitors. To determine whether MTI-101 is synergistic with EGFR inhibitors, PC-9 and HCC4006 cells were treated with varying concentration of both agents, and the combination index was determined using CompuSyn software (Version 1.0.1) A combination index significantly less than 1 is indicative of synergism between the two drugs. Osimertinib and erlotinib were found to have synergy with MTI-101 with the treatment of PC-9 and HCC4006 cells (Figure 6D,E). The PC-9R maintained a synergistic inhibition of growth when MTI-101 treatment was combined with osimertinib (Figure 6F). In contrast, selection for resistance in the H446 (SCLC) line did not result in collateral sensitivity to the standard of care agent cisplatin (Figure 6G). Cisplatin combined with MTI-101 was found to be synergistic in HCC4006, PC-9 and H446 cell lines (Figure 6H). However, cisplatin was only additive in H446-R, indicating that a switch in cell state did not maintain synergy with a DNA crosslinking agent (Figure 6I). Together, these data indicate that, for EGFR-driven NSCLC, alternating treatment between MTI-101 and EGFR inhibitors may be a superior strategy compared to combination strategies. In contrast, a regimen consisting of a cisplatin and MTI-101 combination may yield the optimal outcome based on synergy studies and the emerging MTI-101-resistant phenotype. 

## 4. Discussion

MTI-101 is a cyclic peptide that was developed through the optimization and cyclization of the linear peptide referred to as HYD-1 [6]. The mechanism of action of this novel class of molecules includes the induction of cell death via calcium overload, a finding that was dependent on TRPC1/TRPC4/TRPC5 expression in multiple myeloma cell lines [8]. The cyclized peptide MTI-101 was shown to have activity using castrate-resistant prostate cancer in vivo models [9]. In addition, our laboratory recently demonstrated that selection for resistance to HYD-1, the linear derivative, in myeloma cell lines selected for a compromised cell-adhesion-mediated drug-resistant phenotype (CAM-DR) and that HYD-1 and MTI-101 are more potent in inducing cell death in specimens derived from myeloma patients that have relapsed in therapy compared to newly diagnosed samples [10]. In this paper, we sought to expand on the understanding of the activity of MTI-101 in the context of lung cancer. To better understand the mechanism of action and emergence of resistance, we developed two isogenic drug-resistant lung cancer cell lines. The first line, H446, is a SCLC cell line that is characterized as PTEN-deleted, and contains the overexpression of c-MYC [36,37]. The second cell line is characterized as NSCLC and harbors the exon 19 deletion of EGFR, leading to a constitutive activation of the tyrosine kinase EGFR [34]. In the development of MTI-101-resistant lines, morphological changes were observed in response to the drug treatment. The parental cell lines were more spindle-shaped and consisted of an evenly dispersed monolayer in 2D culture. In contrast, the resistant cell lines display a more cobblestone morphology that clumped to form more cell–cell interactions, along with forming tighter junctions to the culture dishes, supported with the notable increased difficulty in trypsinizing these adherent cells. These characteristics of the resistant cells are consistent with the morphology of more epithelial-like cells that contain tight sheets of cobblestone morphology to form apicobasal polarity and minimize surface energy [38,39]. The change in morphology correlated with the functional inhibition of invasion into Matrigel. It has been shown that transcription factors such as Snail, Twist and nuclear β-catenin regulate the induction of EMT [40] and can lead to increases in invasive capabilities [41,42], as seen in the parental cell lines. Using an unbiased analysis of differential gene expression demonstrated that the hallmark of EMT was reduced in the resistant phenotype. The conversion to a more epithelial-like phenotype was stable as cells removed from the drug for six months retained markers of MET and failed to invade Matrigel. These data suggest a heritable change in cell state, rather than a transient drug-induced state. Importantly, conversion to a more epithelial-like phenotype was observed using two independent in vivo models. In the PC-9 cells, this finding correlated with a delay and reduction in detectable bone metastasis, likely attributed in part to the significantly increased E-cadherin expression. Current clinical trials are targeting EMT in combination with chemotherapies/targeted therapies in lung cancer to reduce drug resistance and metastasis [43]. One example is the repurposing of metabolic inhibitors such as simvastatin for the treatment of lung cancer via the attenuation of TGFß1 [44,45]. In addition, a small molecule drug known as moscatilin has been shown to reverse EMT in lung cancer cells without inducing cytotoxic effects by suppressing mesenchymal markers such as vimentin, Snail and Slug [46]. Despite the breadth of research targeting the EMT pathway in cancer, to the best of our knowledge, the literature has not presented a drug that selectively kills cancer cells in which a MET phenotype emerges as the drug-resistant population. 

Bioinformatic analysis of the RNA SEQ data demonstrated a stable attenuation of expression of genes that are associated with the EMT hallmark in both MTI-101-acquired resistant cell lines. By comparing common differentially expressed genes leading to this negative EMT enrichment, insight can be gained on the cellular remodeling causative for transition toward an epithelial phenotype. One transcription factor that may be driving the MET phenotype with resistance is Twist. The genes Twist1 and Twist2 have both been shown to play a role in the promotion of EMT and invasion in cancer [47,48]. Twist binds to DNA to repress the expression of E-cadherin [49], thereby creating dissociation with β-catenin in the extracellular matrix, causing either the degradation or nuclear localization of β-catenin [40,50]. The activation of the WNT signaling pathway has been shown to activate nuclear β-catenin localization for the induction of EMT, and it is thus intriguing to speculate that WNT signaling may be a determinate of sensitivity to MTI-101-induced cell death [51]. A high expression of Twist1 has also been shown to correlate with the overexpression of fibronectin, vimentin [52] and SPARC [53], which is consistent with some of the most significantly decreased genes in the RNA SEQ data of resistant cells in both cell lines. Fibronectin and vimentin have been extensively studied as mesenchymal markers promoting invasion [20,54,55,56,57]. Fibronectin has also been shown to regulate anoikis resistance by stabilizing cells with the ECM once detached from adjacent cells [58]. A significantly decreased fibronectin expression in both resistant lines could be the cause of increased anoikis-induced apoptosis. There is controversy in the literature on the role of SPARC in cancer [59], but, specifically for lung cancer, it has been shown to be a poor prognostic indicator as it promotes tumor metastasis [60,61]. In contrast, it has also been shown that the loss of E-cadherin itself can induce transcriptional modifications that cause an induction of EMT in cells [62]. It is important to note that MTI-101 has been shown to shift a cell state to a more epithelial-like phenotype, but the EMT process has many intermediate stages of this transition [22]. Further research is needed to determine the heterogeneity of the resistant population and the existence of subpopulations through single cell sequencing to understand if an intermediate stage along this process is involved or if it is a full transformation into MET. Given that Twist was the only EMT transcription factor identified as being downregulated in the MTI-101 drug-resistant cell lines by gene expression profiling, a partial transition to an epithelial state is likely. Importantly, when the drug pressure was removed, the transition state toward MET was stable with respect to the shift in the genotype and phenotype. Clear vulnerabilities were defined, including an increased susceptibility to anoikis and increased sensitivity to EGFR inhibitors. It is intriguing to postulate that Twist levels may be used as a biomarker for the sensitivity of the drug to push cells towards a vulnerable state. The goal of identifying key changes that could be leading to a less aggressive phenotype is to potentially target or modulate cancer cells to reduce the incidence of relapse and prolong survival. 

Patient relapse is almost always inevitable with current standard of care options for metastatic lung cancer. The devastation that comes with drug-resistant tumors is (i) surgical excision of the tumor is not feasible at metastatic sites; and (ii) evasion of drug treatment. MTI-101 may be the first of its kind to induce a more favorable and treatable relapsed tumor. Further studies are required to determine whether the acquisition of resistance to EGFR inhibitors that occurs via an EMT phenotype can be remodeled to an MET phenotype with treatment with MTI-101. Once the MTI-101-resistant cancer cells are given a drug holiday (removed from drug pressure), the shift to an MET phenotype has remained stable for at least six months. This window would be optimal for additional targeting or chemotherapy treatments to eliminate these surviving cancer cells. In lung cancer, resistance to standard of care agents typically yields a more aggressive and drug-resistant EMT phenotype [63,64]. Our findings are similar to what others have shown, such that the reversal of EMT to MET resulted in re-sensitizing cancer cells to tyrosine kinase inhibitors such as gefitinib [65]. The alterations created during MTI-101-acquired resistance enhanced the efficacy of erlotinib and osimertinib in PC-9. Further studies are required to determine whether alterations of drug treatment (i.e., EGFR inhibitor followed by MTI-101 and then a repeated cycle of alternating drugs) would increase survival outcomes in pre-clinical in vivo models of EGFR-driven tumors. 

Overall, drug resistance remains a crucial hurdle in the development of new cancer treatments. Delineating changes that lead to drug evasion and understanding the acquired phenotype give insight into new possible avenues of targeted or combination therapy to optimize patient outcomes. MTI-101 has shown promising anti-cancer activity, along with alterations of a cell state to a more favorable MET phenotype. The acquisition of resistance to MTI-101 has shown unique qualities that have exposed vulnerabilities to the surviving cancer cells. 

## 5. Conclusions

The emergence of resistance to MTI-101 led to genotypic and phenotypic changes that correlated with a MET phenotype using both in vitro and in vivo models in PC-9 and H446 cells. Phenotypic modifications included increased cell–cell connections through morphological changes and increased expression of epithelial-like proteins, while decreasing the expression of mesenchymal-like proteins with a reduction in invasion. The stability of the transformed state in vitro is intriguing and could allow for a double bind therapeutic strategy where the goal is to switch between cell states EMT-MET to keep the tumor from rapidly escaping targeted therapies, such as EGFR inhibitors. Interestingly, the phenotypic selection pressure was survival despite MTI-101 treatment, and this MET population arose concurrently. More studies are required to determine if MTI-101 depletes the EMT population, leading to the emergence of an MET genotype, or, rather, if selecting resistance coincides with the attenuation of calcium signaling and, conversely, if robust calcium signaling is required to support the EMT genotype and phenotype. Resistance to MTI-101 also conferred collateral sensitivity to EGFR inhibitors for PC-9 cells. In addition, MTI-101 combination therapy with standard of care agents erlotinib, osimertinib and cisplatin all displayed synergistic activity in eliminating the cancer cells. These alterations that arise in PC-9 and H446 cells with the sole pressure of MTI-101 suggest a shift to a clinically favorable and long-term mesenchymal-to-epithelial transition that could give insight into novel anti-cancer therapeutic targets and combination strategies to optimize patient outcomes. Future studies are required to determine if following treatment in vivo with a standard of care agent such as erlotinib with MTI-101 and the subsequent emergence of resistance is sufficient to drive the tumor population back toward drug sensitivity and a more epithelial cell state.

## Data Availability

RNA-Seq data were deposited to Gene Expression Omnibus: accession number GSE193455.

## Supplementary Materials

The following supporting information can be downloaded at: https://www.mdpi.com/article/10.3390/cancers14133062/s1, Figure S1: Bioinformatic Analysis of RNA SEQ Predicts a Shift toward a MET Phenotype with Acquired Resistance to MTI-101 in H446 vs. H446-R. EMT genes’ fold change between H446 and H446-R; SPARC-FDR = 33.09 × 10^−4^, VIM- FDR = 6.36 × 10^−4^, FN1- FDR = 2.81 × 10^−4^, TWIST1- FDR = 1.60 × 10^−4^; Figures S2–S4: Original Western blots of TWIST1 *(n* = 3); Figures S5–S7: Original Western blots of TWIST2 (*n* = 3); Figures S8–10: Original Western blots for Figure 4A (*n* = 3); Figure S11: Original Western blot of Figure 4G; Figure S12: H446 In Vivo Single Treatment MTI-101. (A) B-catenin localized heavily inside the cell in the one kidney of the control group with B-catenin presence. (B) A representative image of the treatment group containing B-catenin almost exclusively extracellularly; Figure S13: All H446 In Vivo H&E and IHC Images at 4× Magnification. Kidneys were taken from the H446 in vivo study, fixed in formalin, paraffin blocked and then sliced for slides to analyze EMT markers. Three control and three treatment (10 mg/kg MTI-101) mice were picked at random to compare EMT markers. (A) H&E, (B) E-cadherin, (C) vimentin, (D) B-catenin; Figure S14: Representative IVIS Images of Tumor Location for each In Vivo Study.
