# Supplementary material for: Emergence of Resistance to MTI-101 Selects for a MET Genotype and Phenotype in EGFR Driven PC-9 and PTEN Deleted H446 Lung Cancer Cell Lines
Source: Cancers (Basel). 2022 Jun 22;14(13):3062. doi: 10.3390/cancers14133062 (PMC9264848; doi:10.3390/cancers14133062)
Supplement: Supplementary file 1 [file cancers-14-03062-s001.zip › cancers-1700707-supplementary.pdf]

H446

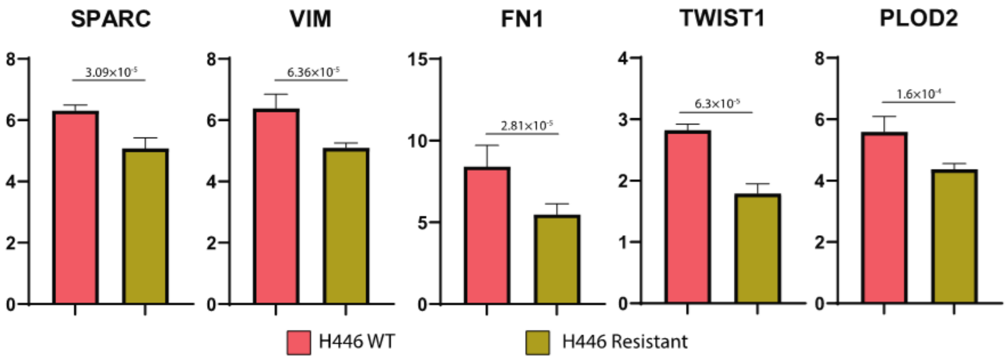

Figure S1. Bioinformatic Analysis of RNA SEQ Predicts a Shift toward a MET Phenotype with Acquired Resistance to MTI-101 in H446 vs. H446-R. EMT genes' fold change between H446 and H446-R; SPARC-FDR = 33.09 × 10^{-4}, VIM-FDR = 6.36 × 10^{-4}, FN1- FDR = 2.81 × 10^{-4}, TWIST1- FDR = 1.60 × 10^{-4}.

Figure 3A (N=2)

S2

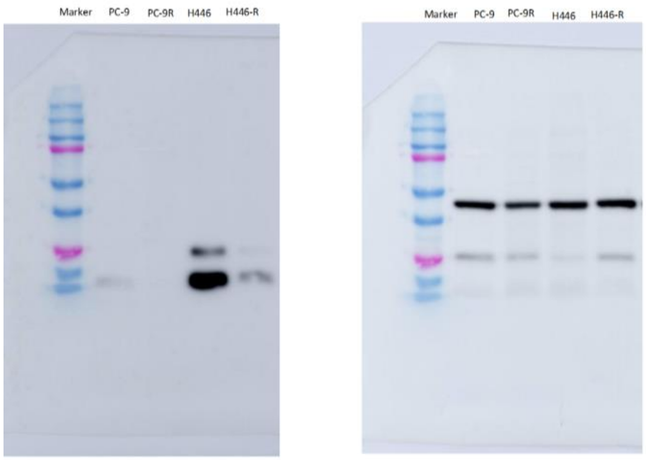

TWIST1 N=2

B-Actin N=2

Figure 3A (N=1)

S3

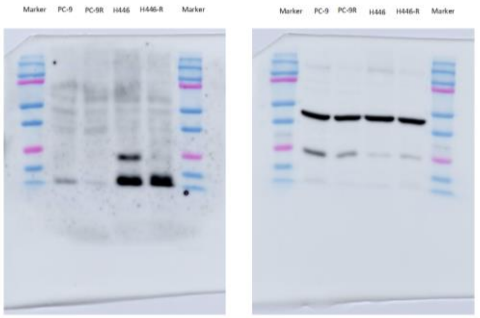

TWIST1 N=1

B-Actin N=1

Figure 3A (N=3)

S4

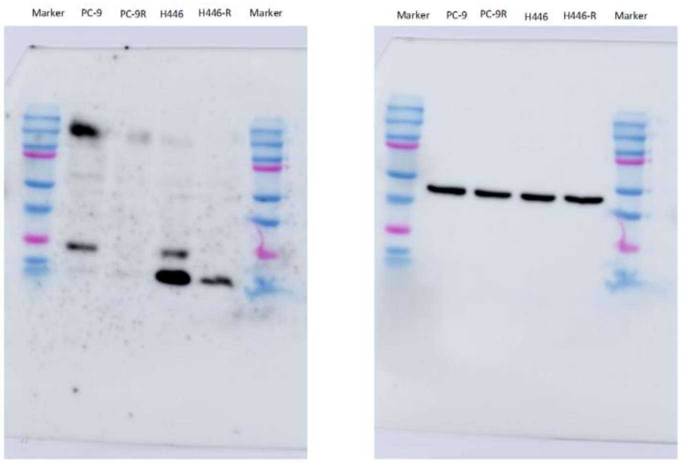

TWIST1 N=3

B-Actin N=3

Figures S2–S4. Original Western blots of TWIST1 *(n* = 3).

Figure 3C (N=1)

S5

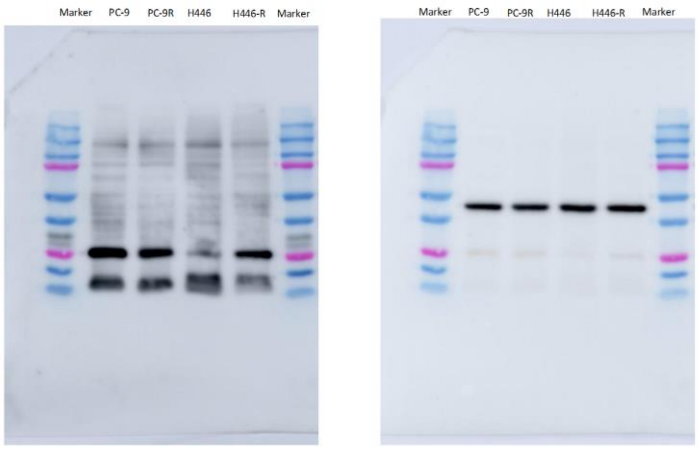

TWIST2 N=1

B-Actin N=1

Figure 3C (N=2)

S6

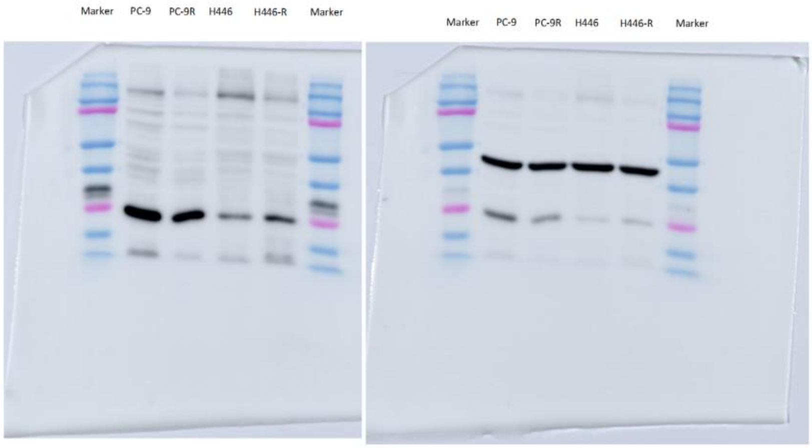

TWIST2 N=2

B-Actin N=2

Figure 3C (N=3)

S7

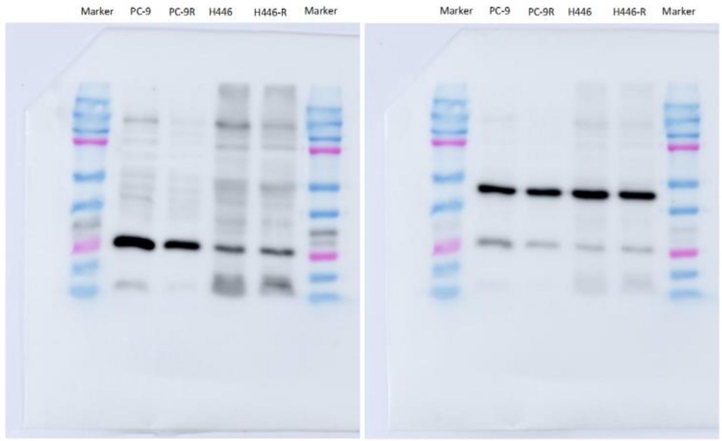

TWIST2 N=3

B-Actin N=3

Figures S5–S7. Original Western blots of TWIST2 (n = 3)

Figure 4A (N=1)

S8

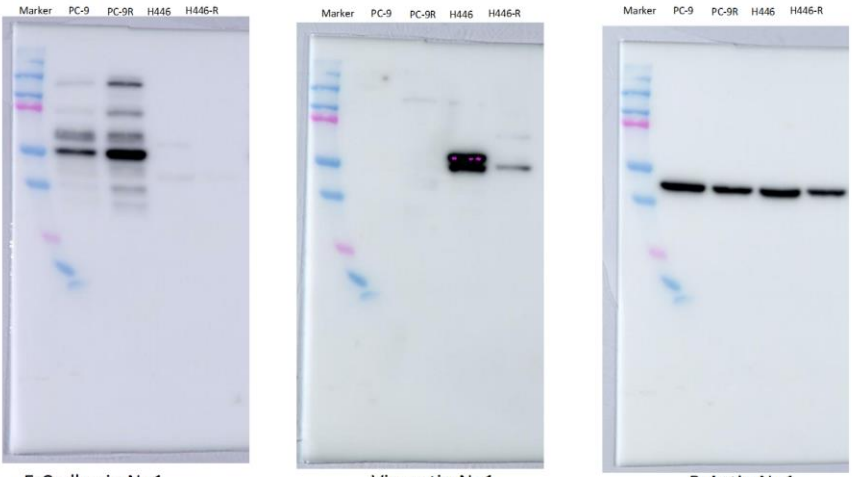

E-Cadherin N=1 Vimentin N=1 B-Actin N=1

Figure 4A (N=2)

S9

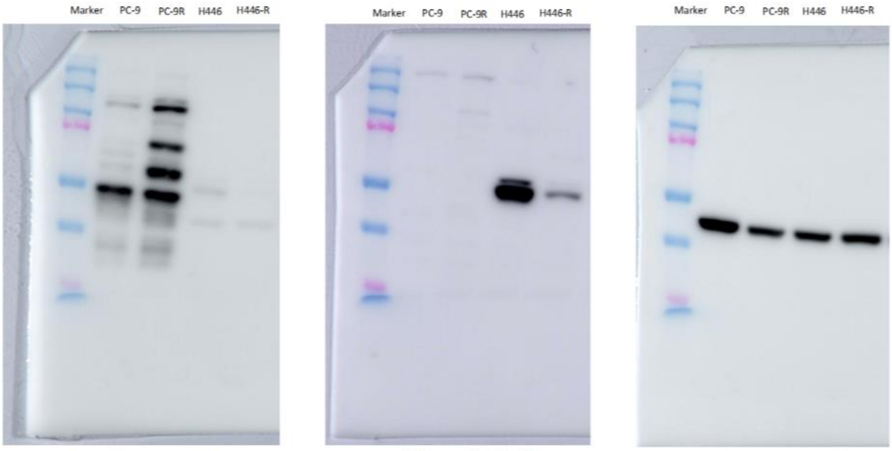

E-Cadherin N=2 Vimentin N=2 B-Actin N=2

Figure 4A (N=3)

S10

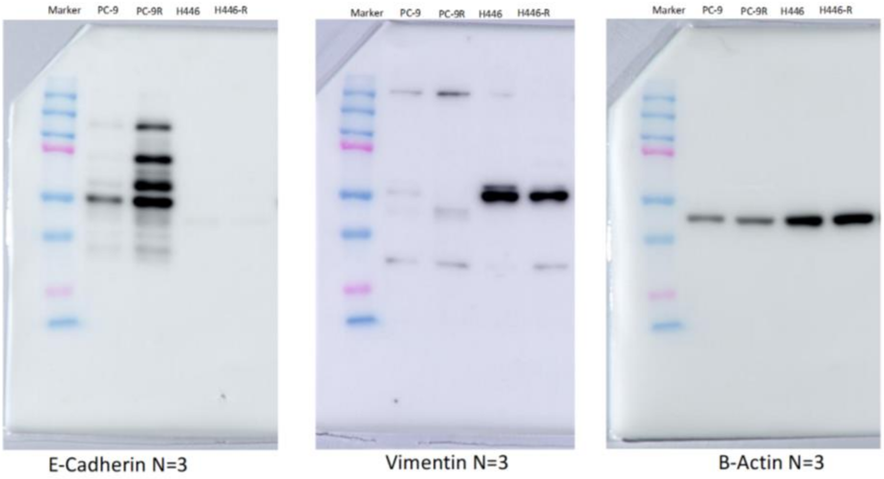

Figures S8–S10. Original Western blots for Figure 4A (*n* = 3)

Figure 4G

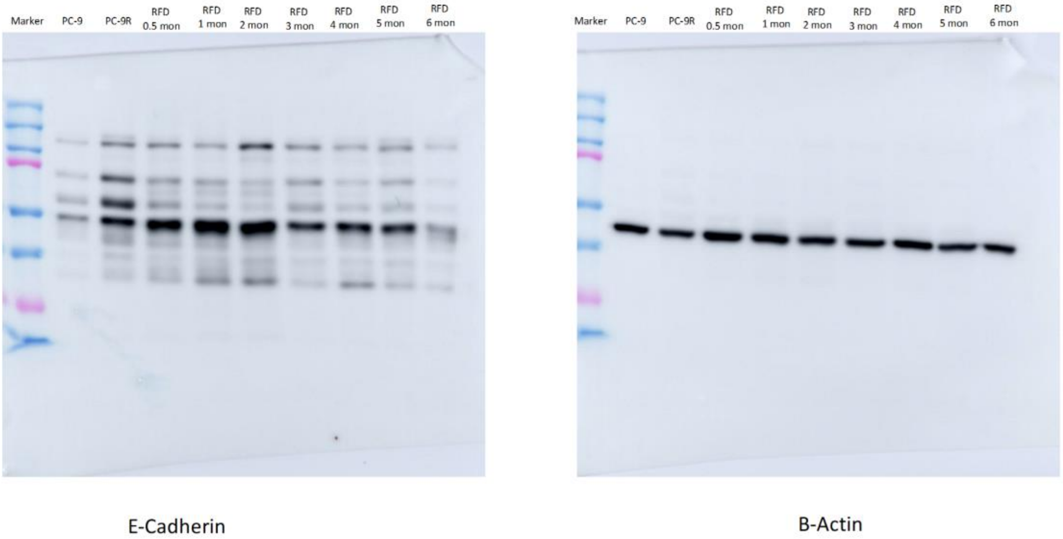

Figure S11. Original Western blot of Figure 4G.

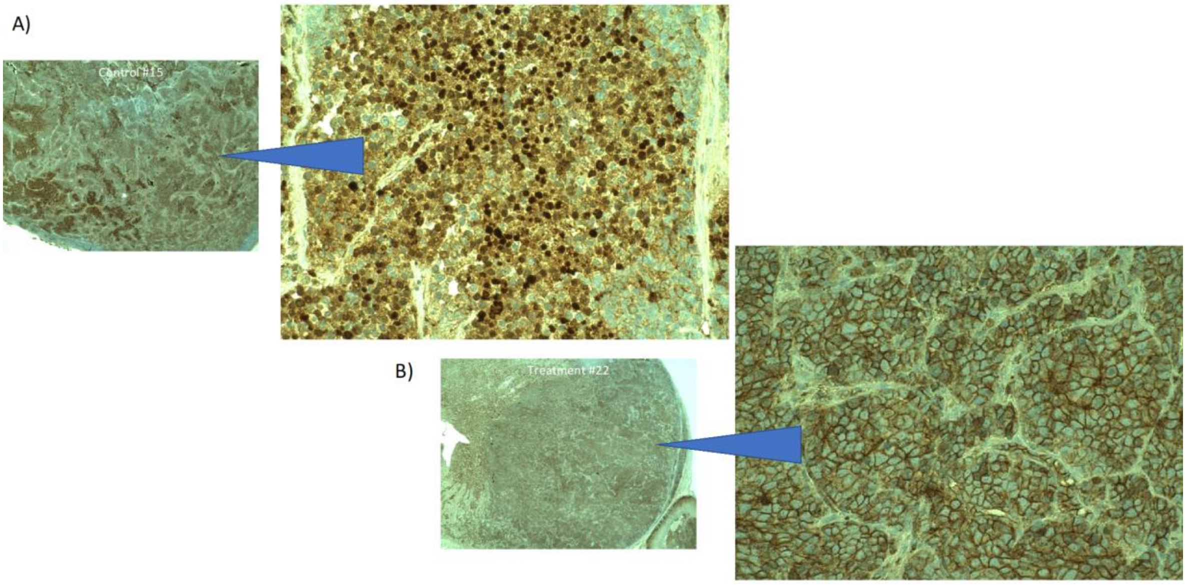

Figure S12. H446 In Vivo Single Treatment MTI-101. (A) B-catenin localized heavily inside the cell in the one kidney of the control group with B-catenin presence. (B) A representative image of the treatment group containing B-catenin almost exclusively extracellularly.

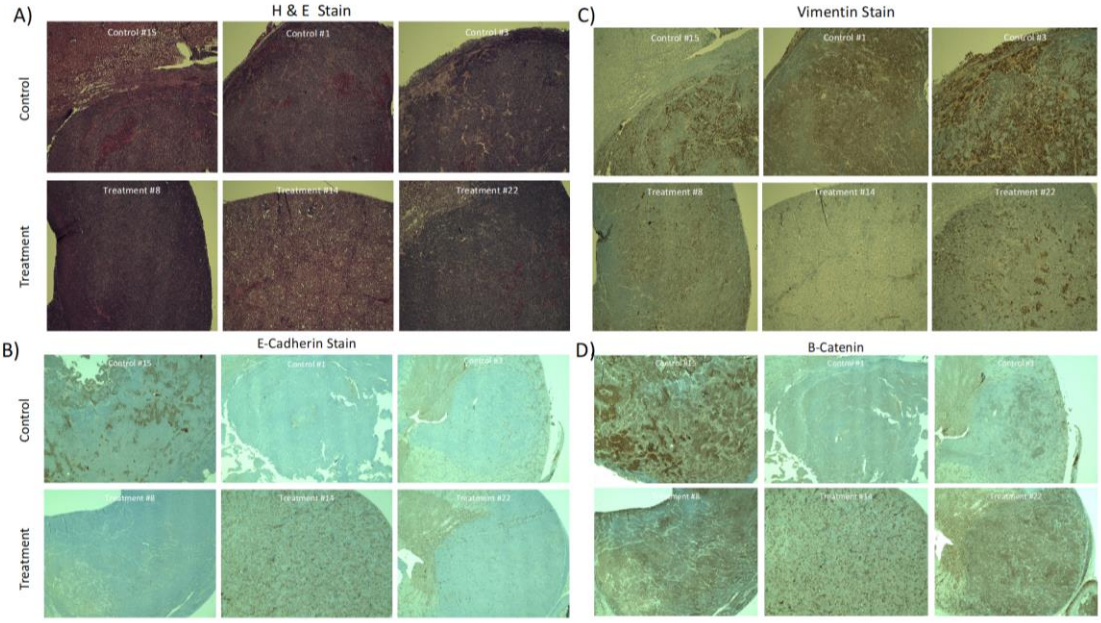

Figure S13. All H446 In Vivo H&E and IHC Images at 4× Magnification. Kidneys were taken from the H446 in vivo study, fixed in formalin, paraffin blocked and then sliced for slides to analyze EMT markers. Three control and three treatment (10 mg/kg MTI-101) mice were picked at random to compare EMT markers. (A) H&E, (B) E-cadherin, (C) vimentin, (D) B-catenin.

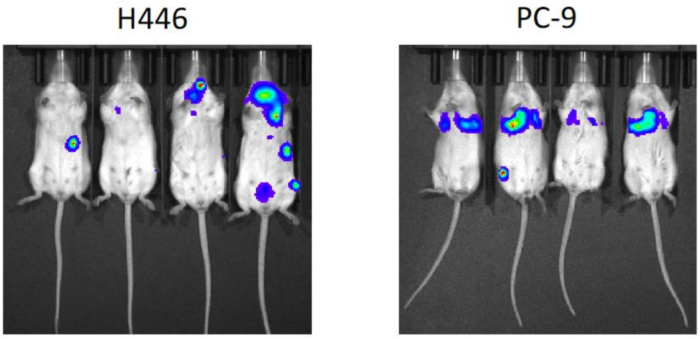

Figure S14. Representative IVIS Images of Tumor Location for each In Vivo Study.
